# High-speed cryo-microscopy reveals that ice-nucleating proteins of *Pseudomonas syringae* trigger freezing at hydrophobic interfaces

**DOI:** 10.1126/sciadv.adn6606

**Published:** 2024-07-03

**Authors:** Paul Bieber, Nadine Borduas-Dedekind

**Affiliations:** Department of Chemistry, University of British Columbia, Vancouver, Canada.

## Abstract

Ice-nucleating proteins (INpro) trigger the freezing of supercooled water droplets relevant to atmospheric, biological, and technological applications. The high ice nucleation activity of INpro isolated from the bacteria *Pseudomonas syringae* could be linked to the aggregation of proteins at the bacterial membrane or at the air-water interface (AWI) of droplets. Here, we imaged freezing onsets, providing direct evidence of these proposed mechanisms. High-speed cryo-microscopy identified the onset location of freezing in droplets between two protein-repellent glass slides. INpro from sterilized *P. syringae* (Snomax) statistically favored nucleation at the AWI of the droplets. Removing cellular fragments by filtration or adding surfactants increased the frequency of nucleation events at the AWI. On the other hand, cultivated intact bacteria cells or lipid-free droplets nucleated ice without an affinity to the AWI. Overall, we provide visual evidence that INpro from *P. syringae* trigger freezing at hydrophobic interfaces, such as the AWI or the bacterial membrane, with important mechanistic implications for applications of INpro.

## Teaser

A high-speed cryo-microscopy technique was developed and applied to visualize proteins from *P. syringae* nucleate ice.

## INTRODUCTION

Ice nucleation is a fundamental process in our atmosphere (*1*), biosphere (*2*), and in many technological applications, such as cryo-preservation (*3*, *4*) and weather modification (*5*). When water droplets reach a supercooled temperature below 0°C, the liquid phase remains metastable until the formation of ice is induced (*6*, *7*). Ice-nucleating substances trigger the formation of ice in atmospheric cloud droplets (*7*–*9*), biologically controlled freezing events (*10*), and artificial snow production (*11*). For example, mineral dusts and organic aerosols can alter the phase state of cloud droplets in the troposphere and therefore influence the global albedo of the planet (*7*, *12*) and its hydrological cycle (*1*). Furthermore, several bacterial species acquire nutrients by triggering freezing of water on plant leaves, which damages agricultural crops (*13*). Yet, exactly which substances are capable of nucleating ice remains difficult to predict due to limited knowledge of the molecular mechanism of heterogeneous ice nucleation.

The bacteria *Pseudomonas syringae* has been isolated from leaf litter and found to nucleate ice at temperatures up to ∼–2°C (*14*–*16*), a high temperature compared to other ice-nucleating substances such as pollen, mineral dust, and some organic particles nucleating ice below −15°C (*17*–*20*). These temperatures are high above the homogeneous freezing temperature of water; supercooled pure water droplets with a diameter of 10 μm freeze within a minute at −38°C (*21*). The nucleation activity of *P. syringae* originates from a specific ice-nucleating protein (INpro) termed inaZ (*22*). According to classical nucleation theory and computational calculations, the templating interfacial area of a single INpro (∼72 nm^2^) is too small to be capable of nucleating ice at high temperatures (*23*, *24*). Aggregates or oligomers of INpro, with larger interfaces (>2000 nm^2^) than a single INpro, are likely responsible for ice nucleation in the high temperature regime (*23*–*27*). Ice-nucleating aggregates consisting of inaZ can be divided into class A, class B, and class C, based on different aggregate sizes triggering freezing at −2°, −6°, and −7°C, respectively (*26*, *28*). Because electrostatic protein-protein and ion-protein interactions likely drive the dimerization and aggregation of INpro, the nucleation ability of INpro oligomers is sensitive to pH fluctuations (*29*, *30*) and the addition of salts (*31*). Furthermore, the bacterial membrane is thought to act as an interface to embed inaZ and control the formation of aggregates (*32*–*34*). However, solutions containing inaZ without the bacterial cells remain ice active (*24*, *33*), suggesting that additional interactions must be involved.

The interface between liquid water droplets and the surrounding air is called the air-water interface (AWI) and is known to be hydrophobic and thus to attract hydrophobic moieties of organic molecules (*35*). To probe intermolecular interactions of INpro, sum frequency generation (SFG) spectroscopy, which is sensitive to interfacial molecules, has been used to analyze the behavior of inaZ molecules and interfacial water at the AWI (*36*–*38*). For example, Pandey *et al.* (*36*) demonstrated that a decrease in temperature led to higher intensities of vibrations associated with ordered H_2_O at the AWI. Furthermore, Roeters *et al.* (*37*) used a similar technique to demonstrate that inaZ reorients at the interface during cooling. SFG and surface tension measurements suggest that INpro are accumulated and activated at interfaces such as the bacterial membrane or the AWI (*36*), however, these measurements do not provide direct evidence that the onset of ice nucleation is at this interface in a spherical droplet. A study using heat-inactivated INpro that lost its ice nucleation ability showed a similar structural ordering of water molecules at the AWI, further raising questions about the role of the ordering (*38*). Moreover, SFG spectroscopy probes a relatively large flat area of the AWI above the actual freezing point of the solutions, assuming that the curvature of smaller droplets and temperatures closer to the freezing point do not influence the behavior of INpro at the AWI. D_2_O is often used to analyze INpro at the AWI to avoid the infrared absorption overlap between H_2_O and amides in INpro solutions (*39*). However, intermolecular forces of D_2_O deviate from H_2_O (*40*), thereby potentially altering the structure of proteins in solutions (*39*), which could, in turn, influence the ice nucleation mechanism of INpro. To date, microscopic observations of heterogeneous ice nucleation at the AWI induced by INpro are missing.

Direct visualizations of ice nucleation events at a specific location or interface remain challenging. Once an ice embryo forms in a supercooled droplet, the ice phase propagates quickly. It takes less than 100 ms to turn a 1-μl droplet into ice, depending on the temperature (*41*). For most drop freezing techniques, only binary data are obtained, where the droplet is either liquid or frozen at a given temperature [examples of optical freezing assays in (*42*–*45*)]. This binary type of detection cannot resolve information about the nucleation’s origin.

To capture the location of initial freezing, high-speed cameras have recently been used to study ice nucleation on mineral dust, aluminum plates, and salty aqueous solutions (*46*–*49*). Holden *et al.* (*46*) located the origins of ice crystal growth on feldspar and quartz surfaces by taking videos with 3000 frames per second during the freezing process. Another study used a high-speed camera to analyze heterogeneous ice nucleation induced by a macroscopic aluminum plate at the three-phase contact line between aluminum, water, and air or oil (*48*). Furthermore, high-speed videos have been used to identify the onset of crystal growth at the AWI for acoustically levitated droplets (*49*). Here, we developed a method using high-speed imaging to analyze the ice nucleation activity of macromolecules at interfaces and in the bulk of a supercooled droplet. With this method, we visualized the ice nucleation location of INpro at hydrophobic interfaces for the first time.

## RESULTS

### Development of the experimental setup

**Fig. 1.**
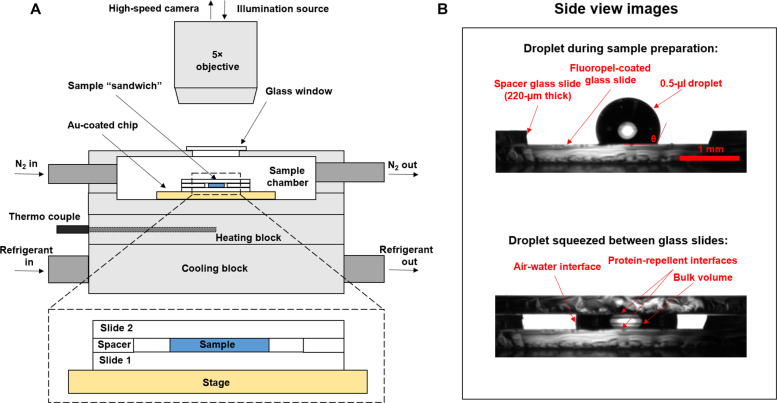
Instrumentation of the cryo-microscope equipped with the high-speed camera. (**A**) Schematic drawing of the technical setup and (**B**) images of a droplet during sample preparation and squeezed between two glass slides. Image (B) is intended to be a real image of the dotted box in (A) and was taken with the optical sample stage of the tensiometer.

**Fig. 2.**
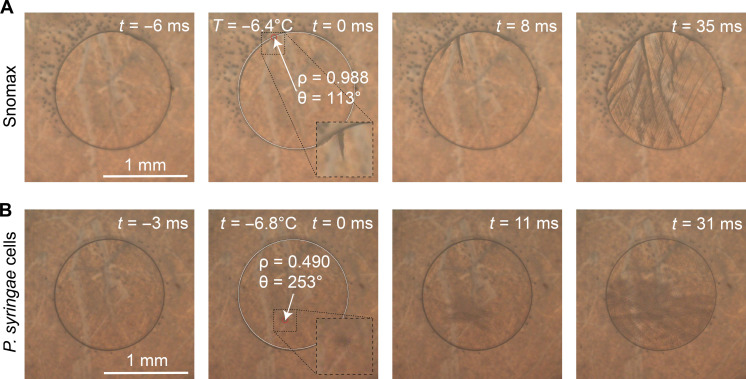
Example images of high-speed freezing experiments of aqueous samples. (**A**) Snomax (10^−3^ wt %) and (**B**) *P. syringae* cells. The frame in which the nucleation event was detected was set to 0 ms. A white arrow indicates the polar coordinates obtained from the measurement, and an inset shows a zoomed-in image of the forming ice-dendrites. Time-stamped images before and after the initial freezing events are shown with different time marks. See additional images in figs. S7 and S10 and at https://doi.org/10.5281/zenodo.8277977.

Building upon three studies that used high-speed cameras for ice nucleation research (*46*, *48*, *49*), we developed an experimental setup for detecting the onset location of nucleation in droplets containing organic ice-nucleating substances. Specifically, we combined a high-speed camera with a cryo-microscopic instrument to detect ice nucleation at the AWI of a pancake-shaped aqueous droplet (Fig. 1). In our experiment, the sample solution was pipetted onto a glass slide and sandwiched between a second glass slide at a height of 220 μm, to visualize the AWI around the circumference of the droplet from a microscopic top view (Fig. 2). This assembly allows the projection of the AWI of the droplet in two dimensions (Fig. 2). The glass slides were coated with a protein-repellent fluorinated polymer to minimize the adsorption of proteins (*50*, *51*), thereby reducing INpro interactions with the glass slides. The role of glass slide-INpro interactions was thoroughly assessed and is discussed later. Our high-speed cryo-microscopy technique records videos of freezing droplets with more than 2000 frames per second and enables us to locate the freezing onset and temperature of INpro solutions (Fig. 2). The accuracy of the recorded temperatures (fig. S1) was validated with comparison measurements between other ice nucleation instruments (fig. S2). Freezing temperatures obtained with the drop Freezing Ice Nuclei Counter (FINC), characterized by Miller *et al.* (*42*), overlap with the recorded temperatures of the cold stage (fig. S2), thereby confirming accurate readings (±0.5°C).

### INpro from Snomax nucleate ice at the AWI

To observe whether INpro nucleate ice at the AWI as proposed by several studies (*26*, *36*, *37*), we studied the nucleation behavior of samples containing INpro in our setup. The commercially available substance called Snomax, used for artificial snow production, is produced from cultivated *P. syringae* bacteria, treated with centrifugation, freezing, lyophilization, and sterilization procedures (*52*). The product consists mainly of proteins, carbohydrates, and nucleic acids, but the exact composition and preparation of Snomax remain proprietary (*52*). Its ice nucleation activity has been attributed to aggregates of inaZ, mainly attached to cellular membrane fragments (*11*, *53*). We refer to all forms of inaZ aggregates and inaZ attached to bacterial fragments in Snomax collectively as INpro from hereon. Note that filtrates of Snomax (cutoff at 0.2 μm) remain ice active (*54*), indicating the presence of INpro free from bacteria cells. Aqueous droplets containing Snomax (10^−3^ wt %) were analyzed in the high-speed setup. Thanks to the high time resolution of the camera, the onset location of the ice crystal could be located once ice started to propagate from the AWI through the droplet (Fig. 2A).

**Fig. 3.**
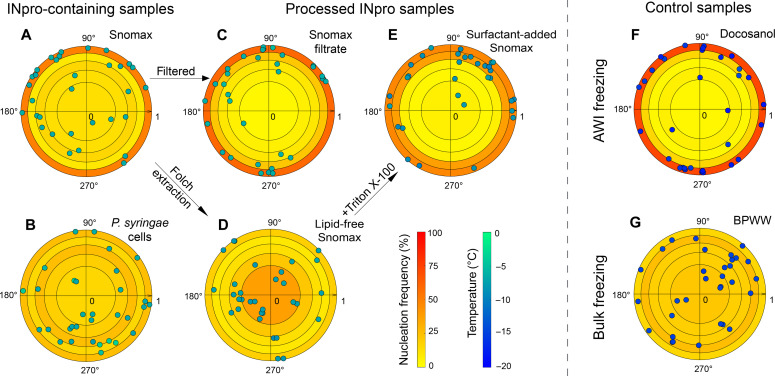
Onset nucleation locations, frequencies, and temperatures shown as target plots for INpro-containing, processed INpro and control samples. Every nucleation event that was localized from the droplet was converted to polar coordinates and to a temperature value. INpro-containing samples (**A**) Snomax (10^−3^ wt %, surface tension = 72.1 ± 0.2 mN m^−1^) and (**B**) cultivated intact *P. syringae cells* are shown on the left. Processed INpro samples (**C**) filtered Snomax, (**D**) Folch extracted Snomax protein fraction (lipid-free Snomax, surface tension = 72.0 ± 0.5 mN m^−1^), and (**E**) Folch extracted Snomax protein fraction with added Triton X-100 (surfactant-added Snomax, surface tension = 33.2 ± 0.2 mN m^−1^) are shown in the middle. Control samples (**F**) docosanol and (**G**) birch pollen washing water (BPWW) are shown on the right. The color bar from green to blue indicates the freezing temperatures, whereas the color bar from yellow to red indicates the nucleation frequency in each volume.

To gain statistically significant results, we conducted the experiment 32 times with eight different droplets (i.e., each droplet was frozen four times; figs. S3 and S4). Details on the choice of replicates, number, and the use of Monte Carlo simulations are in the Supplementary Materials. We concluded that 32 replicates unambiguously allowed (i) the distinction between freezing from the bulk and the AWI and (ii) the compromise for a reasonable number of lab experiments (figs. S5 and S6). The comparison of the freezing temperatures with the FINC instrumentation confirms that 32 datapoints are representative for a measurement with 288 datapoints (fig. S2). All freezing events were above the blank freezing range of −23.2 to −30.0°C and the median freezing temperature (*T*_50_) of −25.8°C (fig. S1), confirming that we are observing INpro nucleating ice and not background contamination. We pooled the 32 observations and analyzed the nucleation frequency within five equivalent volumes of the pancake-shaped droplet in concentric circles (see fig. S5). We normalized the radii of the frozen droplets to 1 and plotted the polar coordinates of the freezing events with the corresponding temperatures (Fig. 3). The nucleation frequency in the outermost sector of the pancake-shaped droplet was used to elucidate the role of the AWI in the ice nucleation mechanism. Because the size of the outermost sector is a fifth of the volume, the expected nucleation frequency in this area is 20%. To quantify the statistical variation of our experimental design, we conducted Monte Carlo simulations (*55*) and found a mean nucleation frequency of 20% in the outermost segment with an SD of 7.2% when analyzing 32 randomly distributed nucleation onset locations (fig. S6). Therefore, we confirm that the ice nucleation mechanism, of samples that nucleate in the outermost sector more often than in 42% of the observations [mean (20%) plus three times the SD (7.2%)], is statistically influenced by the AWI. Ice nucleation at the AWI influences the distribution of nucleation sites within the analyzed droplet containing Snomax (10^−3^ wt %), because 50% of the nucleation events were observed in the outermost sector (Fig. 3A).

Although there is a clear preference for INpro to nucleate at the AWI (Fig. 3A), there were experiments where nucleation occurred in the bulk of the pancake-shaped droplet (fig. S7). We wondered whether a freeze-thaw cycle was affecting the ice nucleation sites of Snomax similar to (*56*), but there was no drift in nucleation temperatures or a trend in the locations over the four freeze-thaw cycles (figs. S3 and S4). This result indicates that the Snomax sample remained stable during our freeze-thaw experiments and that the freezing mechanism did not change from one cycle to the next.

### Concentration and temperature dependency

To investigate whether the nucleation frequency at the AWI depends on the concentration of the sample, we measured Snomax in two additional mass concentrations (10^−1^ and 10^−5^ wt %). The *T*_50_ values were −4.1°, −6.5°, and − 9.0°C for samples with the mass concentrations of 10^−1^, 10^−3^, and 10^−5^ wt %, respectively (fig. S1 and table S1). To further characterize surface active molecules at the AWI, we measured the surface tension of Snomax solutions with a pendant droplet tensiometer. The surface tension decreased from that of pure water at 72.2 ± 0.2 (SD) mN m^−1^ down to 56.2 ± 0.9 (SD) mN m^−1^ for the most concentrated sample (table S1). This observation is consistent with the presence of surfactants at the AWI. Yet, a decrease was not observed for the more diluted Snomax aqueous samples, implying that fewer surfactants are at the AWI (table S1), despite ice nucleation occurring at the AWI (fig. S8). Surfactants lower the surface tension when concentrated at the AWI, but the ice nucleation mechanisms depend on the chemical structure of the ice-nucleating molecules (*57*). Therefore, the surface tension cannot be used to predict whether a sample will nucleate ice at the AWI. This conclusion supports the need for direct microscopic observations using our high-speed cryo-microscopy technique.

We found that INpro induced freezing in the outermost sector of the droplet more often than in all other sectors, at all analyzed concentrations (additional images in fig. S7). The nucleation frequency was 50, 50, and 44% in the outermost sector for the Snomax samples with the concentrations 10^−1^, 10^−3^, and 10^−5^ wt %, respectively, clearly above the expected value for bulk nucleation plus three times the SD (Fig. 3A and fig. S8). This trend supports literature data stating that INpro of Snomax and water molecules are orientated at the AWI (*26*, *36*, *37*). Also, we did not notice a temperature dependency for nucleation at the AWI in the investigated concentration range of Snomax (fig. S9). Therefore, we exclude that INpro from Snomax nucleate ice at the AWI and in the bulk at substantially different temperatures and, furthermore, hypothesize that there is only little or no concentration dependency. Overall, we identified a statistically significant increase of ice nucleation at the AWI for Snomax over a large range of concentrations, suggesting that INpro from different types (classes A, B, and C) increase the ice nucleation ability at the AWI of droplets.

### Control experiments with nonpolar and polar ice nucleators

Next, we aimed to prove that the observed nucleation trend at the AWI is a characteristic behavior of INpro and depends on the nature of the ice-nucleating substance. We conducted control and comparison experiments by measuring the onset nucleation location, frequency and temperature of docosanol and birch pollen extracts, as examples of nonpolar and polar ice nucleators, respectively (*17*, *58*–*60*).

Docosanol is a long-chain alcohol that is insoluble in water and forms an ice-nucleating monolayer by assembling its hydrophobic alkyl-chain at the AWI of droplets (*58*–*60*). Therefore, water droplets coated with docosanol should serve as a positive control for ice nucleation at the AWI. Indeed, the docosanol sample showed nucleation at the outermost sector of the droplets in 72% of the analyzed freezing experiments (Fig. 3F; see an example in fig. S10A). The *T*_50_ value was −17.1°C, consistent to literature values (*58*–*60*).

In addition, we measured the surface tension of a droplet coated with docosanol with a tensiometer, to further quantify the surface tension reduction by docosanol. The surface tension reduced from 72.2 ± 0.2 (SD) mN m^−1^ to 58.7 ± 3.2 (SD) mN m^−1^ by adding docosanol, indicating the formation of an organic layer at the AWI (table S1). The ice nucleating ability and the decrease in surface tension confirm that our setup behaves as expected with a known ice-nucleating surfactant.

Birch pollen washing water (BPWW) is an aqueous extract from *Betula pendula* pollen, commonly used as standard for ice nucleation experiments (*17*, *61*–*65*). The chemical identity of the ice nucleator in BPWW remains elusive, but several studies have suggested that a polysaccharide or a glycoprotein might be responsible for its ice nucleation ability (*61*, *63*, *65*). There is no evidence for interactions of ice nucleators of BPWW with hydrophobic interfaces or cellular membranes in the literature. To further show that freezing at the AWI is specific for a certain ice nucleation mechanism, we measured the ice nucleation activity and location of a droplet containing BPWW. As expected, the growing ice dendrites appeared separated from the AWI, clearly demonstrating nucleation without affinity for the AWI (fig. S10B). The *T*_50_ value of −14.8°C agrees with literature data (*17*, *62*, *64*). Overall, we detected freezing events in all sectors of the droplet (Fig. 3G), with 84% of the freezing events originating in the inner sectors, indicating ice nucleation in the bulk.

In contrast to the docosanol sample, the surface tension of the BPWW sample was similar to the blank water [72.2 ± 0.5 (SD) mN m^−1^], suggesting no enrichment of macromolecules at the AWI of the droplets (table S1). The random distribution of onset locations across the volume of the droplets (Fig. 3G) indicates that our setup is capable of identifying bulk ice nucleators.

### Bacterial membranes and lipids influence the nucleation frequency at the AWI

Hypothetically, INpro could be binding to cellular fragments that contain bacterial membranes and trigger freezing in the bulk (*33*). We therefore tested the influence of the cellular membrane and associated lipids of *P. syringae* on the nucleation onset location.

### Effects of bacterial membranes and its fragments

First, we investigated why Snomax was nucleating at the AWI sector of the droplet less often (50%; Fig. 3A) than the docosanol control sample (72%; Fig. 3F). We filtered the most concentrated Snomax sample and measured the location of nucleation (Fig. 3C). The frequency of nucleation in the outermost sector increased from 50 to 59% (Fig. 3C). This increase is above one SD (7%), determined by Monte Carlo simulations (fig. S6), and may support the role of the membrane fragments on bulk nucleation. In addition, the median nucleation temperature decreased from −4.1° to −6.3°C, indicating that the removal of large aggregates and cellular fragments from Snomax may be leading to a transition from class A to class C ice nucleation (*24*, *26*). The increase of nucleation at the AWI and the decrease in nucleation temperatures suggest that nucleation events, which do not originate at the AWI, are likely induced by large aggregates and fragments in the volume of the droplet. Larger surfaces provide nucleation templates for higher onset temperatures (*60*) and, therefore, overshadow freezing events at lower temperatures, caused by smaller oligomers of inaZ at the AWI.

To support this claim, we cultivated *P. syringae* cells and measured a sample containing intact cells with the high-speed camera setup (Fig. 2B). According to Lukas *et al.* (*26*) and our own running hypothesis, a sample with an intact cellular membrane can organize inaZ proteins. Our measurements show that nucleation in the outermost sector only occurred in 25% of the investigated samples (Fig. 3B), indicating no preference for INpro of intact *P. syringae* cells to freeze at the AWI. Instead, the intact bacterial membrane provides a large surface to anchor INpro and triggers freezing in the bulk.

### Effects of lipid removal and surfactant addition

**Fig. 4.**
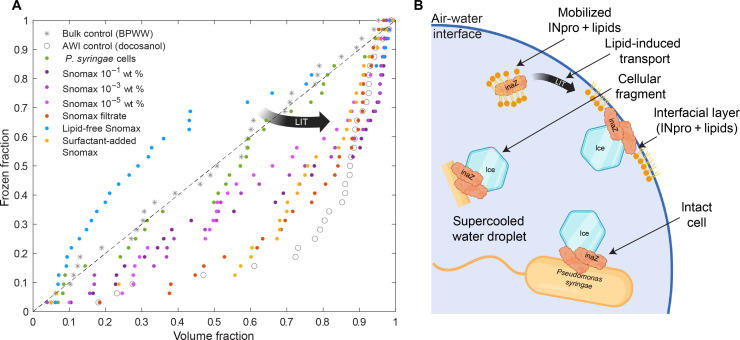
Ice nucleation onset locations depend on the freezing mechanism. (**A**) The fraction of frozen droplets is plotted against the volume fraction at the position of the freezing event. The volume fraction of 0 represents the middle of the droplet, whereas volume 1 represents the AWI. Docosanol serves as a control for ice nucleation at the AWI (gray hollow circles) and BPWW as a control for volume nucleation (gray stars). Snomax in different mass concentrations is displayed as purple and filtered Snomax as orange. Lipid-free Snomax prepared by the Folch extraction is shown in blue, and the same sample with added surfactants (Triton X-100) is shown in yellow. The cultivated *P. syringae* sample is shown in green. A theoretical dashed line representing bulk nucleation is drawn in black. (**B**) A schematic drawing of the proposed ice nucleation mechanisms based on illustrations from (*26*, *37*) was created with BioRender.com. The lipid-induced transport (LIT) is depicted in both figures.

Furthermore, we investigated the effect of lipids on the ice nucleation location of Snomax. We removed lipids from the proteins of Snomax via the Folch extraction procedure as described by Schwidetzky *et al.* (*33*). We measured the surface tension of the extracted sample to be similar to pure water [72.0 ± 0.5 (SD) mN m^−1^], indicating the efficient removal of surfactants, including lipids (table S1). The nucleation temperature of the sample decreased from −4.1° to −7.6°C after the membranes and lipids were removed (Fig. 3D and table S1). The decrease in the freezing temperature indicates that large aggregates bound to membrane fragments are disassembled, reducing the ice nucleation activity of the sample (*33*). The nucleation frequency at the AWI decreased from 50 to 19%, indicating that the nucleation mechanism transitioned from nucleation at the AWI to nucleation in the bulk (Fig. 3D). Because membrane proteins rely on lipids or surfactants to dissolve in water (*66*), the removal of the lipids through the Folch extraction could have resulted in the production of non-soluble aggregates. These aggregates could then be suspended in the bulk of the droplet and cause nucleation in the bulk. In other words, removing the lipids from the sample interfered with the lipid-induced transport of INpro to the AWI interface of the droplet (Fig. 4).

Next, to test the influence of surfactants on the system, we added Triton X-100 to the Folch-extracted sample. Triton X-100 is a nonionic surfactant that solubilizes membrane proteins and sustains their function, i.e., tertiary structure (*67*). We measured a decrease of the surface tension to 33.2 ± 0.2 mN m^−1^ when the surfactant was added (table S1). The addition of surfactant further decreased the nucleation temperature to −8.7°C and reinstated nucleation at the AWI with a frequency of 47% of the 32 measurements (Fig. 3E). This result confirms that surfactants can effectively dissolve membrane proteins by forming a micelle-INpro complex corroborating Rawlings’ work (*66*). Thus, INpro transported by Triton X-100 micelles accumulated at the AWI and froze at the AWI (Fig. 3E). Our results indicate that ice nucleation at the AWI interface is influenced by surfactants (following Fig. 3, A to D to E).

We conclude that the surface tension cannot quantitatively predict the nucleation frequency at the AWI. For example, the diluted Snomax sample (10^−3^ wt %) did not reduce the surface tension [72.1 ± 0.2 (SD) mN m^−1^] and nucleated ice at the AWI sector in 50% of the cases, whereas the surfactant added sample had a surface tension of 33.2 ± 0.2 (SD) mN m^−1^ and nucleated ice at the AWI in 47% of the investigated freezing events (table S1). The surfactants influence the transport of INpro to the AWI; however, the nucleation location is a result of INpro-surfactant-AWI interactions (Fig. 4) and does not solely depend on the surfactant concentration.

### Comparison of the INpro distributions

To quantitatively compare the preference for the AWI between the samples, we plotted the fractions of frozen droplets for all Snomax samples, *P. syringae* cells, docosanol, and BPWW against the squared normalized radius of the nucleation location, equivalent to the volume fraction of the pancake-shaped droplet (Fig. 4A). In the case of bulk nucleation, the frozen fraction should theoretically be a linear function of the volume (Fig. 4A, dashed line). We see precisely this behavior for BPWW (Fig. 4A, gray stars). On the other hand, if the nucleation mechanism is influenced by the AWI, then the frozen fraction will deviate from the linear behavior toward the outermost volume, specifically toward the 0.8 to 1.0 volume fractions. Theoretically, a process purely at the AWI would be represented in Fig. 4A as a vertical line at the value of 1.0 on the *x* axis. Droplets coated with docosanol show the most affinity for nucleation at the AWI, as expected (Fig. 4A, hollow circles).

The frozen fraction values for Snomax fall between the data points for docosanol and BPWW (Fig. 4A). All different concentrations of untreated Snomax show a clear deviation from the linear correlation between volume and frozen fractions, due to the ability of INpro to nucleate ice at the AWI. There may be a trend where the more concentrated samples are more likely to nucleate ice at the AWI than the less concentrated sample (fig. S8). Bulk nucleation events can be found for all unfiltered Snomax samples between a volume fraction of 0.0 to 0.8 (Fig. 4A, purple circles). However, filtering the sample shifts the curve toward the AWI region (Fig. 4A, orange circles). Unfiltered solutions contain cellular fragments with INpro freezing in the bulk, whereas the filtration of Snomax enhances nucleation at the AWI via an interfacial layer of INpro and lipids (Fig. 4B).

Removing lipids and membrane fragments from the Snomax sample led to the insolubilization of INpro (Fig. 4A, blue dots). Following the Folch extraction, the INpro aggregates lost their affinity to the AWI. The deviation from the linear behavior across the volume is probably caused by the formation of large insoluble particles that are not randomly distributed across the volumes of the droplets. Some of the larger particles might have settled at the bottom of the glass slide before the droplet was sandwiched, causing more nucleation events in the middle of the droplet. The heterogeneous distribution of particles in the suspension results in the frozen fraction graph deviating from the linear behavior observed for BPWW (Fig. 4A, gray stars). However, the addition of a surfactant to this sample restored the AWI affinity of INpro (Fig. 4A, yellow circles). This behavior demonstrates that the process leading to nucleation at or close to the AWI depends on the matrix of the sample and the properties of INpro.

Last, freezing locations of the *P. syringae* sample linearly correlate with the corresponding volume fraction, similar to the BPWW control sample (Fig. 4A, green circles), due to the high abundance of intact cellular membranes. Therefore, we can confirm that an intact cellular membrane orientates inaZ in this sample and freezing occurs in the volume in which the intact cells are suspended, as schematically drawn in Fig. 4B. Clearly, anchored INpro are distinguishable from INpro that are free to diffuse to the AWI (Fig. 4).

### Exchanging glass interfaces in the setup

#### Glass slide coating

Furthermore, the fluoropel-coated glass slides can potentially adsorb INpro, which could skew the interpretation of ice nucleation events in the bulk in our experiment. However, because of the protein-repellent properties of fluorinated surfaces (*50*, *51*), this pathway is thought to be of minor influence. To showcase how the experiment is influenced by the fluoropel coating, we measured the filtered Snomax sample on a siliconized glass slide with a lower hydrophobicity and oleophobicity in comparison with the fluropel slide (fig. S8). The nucleation frequency in the outermost sector decreased from 59 to 38%, while the freezing temperature remained the same (*T*_50_ = −6.3°C). This observation suggests that the less repellent siliconized glass slide served as a substrate for INpro to nucleate ice on and confirms that glass slide-INpro interactions are possible but reduced to a minimum in our experimental setup with fluoropel that can be considered to be a protein-repellent coating.

#### Oil-water interface

In addition, we investigated how INpro behave when we replace the AWI with a hydrophobic oil interface. We suspended a droplet of the filtered sample in an inert matrix of halocarbon oil. The emulsion was squeezed between the glass slides resulting in a cylindrical droplet entirely surrounded by oil (example images in fig. S7). The freezing temperature of the droplets did not change in comparison with the sample surrounded by air and yielded a *T*_50_ value of −6.3°C, indicating that the ice nucleation activity is not suppressed when the AWI is excluded. Therefore, we propose that adsorption and accumulation of INpro can take place on different hydrophobic interfaces, which also explains why INpro are ice active in water-oil emulsions (*54*, *56*), including fluorinated oil (*44*). The oil phase led to an even distribution of nucleation events because a hydrophobic layer covered the full area surrounding the droplet. Overall, this experiment supports the proposed hypothesis; INpro sorb to hydrophobic interfaces to nucleate ice. Our results also indicate that the ice nucleation ability of inaZ is not limited to the AWI or membranes but rather more broading due to hydrophobic-aqueous interfaces.

#### Ice crystal morphology and growth velocity

In addition to the detection of the nucleation location and temperature, the developed high-speed cryo-micropscope allowed us to study the morphology of propagating ice crystals and their growth velocity. First, we identified that the ice phase propagates in the form of large dendrites when the nucleation temperature is above −8°C, as seen for Snomax (Fig. 2A). At lower nucleation temperatures, below −15°C, the propagation of the ice-phase is faster, resulting in the formation of a homogeneous ice-crystal front (fig. S10). These findings are consistent with other studies investigating propagating ice crystals (*41*, *68*) and indicate that the difference in crystal morphology in our experiments is caused by different freezing temperatures. Furthermore, we quantified the growth velocity of the ice phase from high-speed videos (figs. S11 and S12). The propagation velocity correlated with the supercooling temperature in the investigated temperature range between −17.2° and −4.1°C and was around 5.9 cm s^−1^ for a supercooling temperature of 10.2°C (fig. S12). These findings indicate that the crystals are growing in the non-diffusion–limited regime (*69*) and that there is no interaction between glass slides and growing ice dendrites (*41*, *68*).

## DISCUSSION

We used high-speed cryo-microscopy to visualize the ice nucleation onset location triggered by INpro at hydrophobic interfaces, such as the AWI of supercooled aqueous droplets (Fig. 2A) and the bacterial membrane (Fig. 2B). The application of high-speed cameras to study supercooled droplets allowed us to visualize for the first time the AWI for ice nucleation induced by organic matter in single droplet experiments. We show unambiguously that the fraction of freezing events of INpro from Snomax is higher at the AWI sector than in the bulk of the droplet (Fig. 3A). The affinity of INpro to sorb to the AWI is influenced by lipids in the sample matrix (Fig. 3, D and E). Removal of larger fragments increased the affinity for the AWI (Fig. 3C). In contrast, INpro on intact *P. syringae* cells nucleate in the bulk of supercooled droplets (Fig. 3B). Our data support the proposed mechanisms of the bacterial protein inaZ needing to be accumulated at an interface to nucleate ice (Fig. 4B), thereby corroborating the proposed molecular mechanisms put forward by Pandey *et al.*, Lukas *et al.*, Hartmann *et al.*, and others (*24*, *26*, *29*, *36*–*38*).

### Limitations

This study used the commercially available product Snomax for investigating its ice nucleation mechanisms. To account for the complex sample matrix, we separated proteins from lipids and polar molecules (Fig. 3) and clearly showed the effect of surfactants on the ice nucleation location. Investigations of purified inaZ proteins (*24*, *70*) and modification of the surfactant concentrations could provide further insights in the nucleation mechanisms at the AWI.

The applied methodology did not allow us to distinguish between ice nucleation events involving the outermost layer of water molecules at the AWI and nucleation events that initiate close to the AWI. The environment of water close to the AWI is characterized by lower densities and stronger electric fields, which could increase the ice nucleation activity (*71*). Molecular dynamic simulations of heterogeneous freezing could identify the role of water molecules at the AWI for the ice nucleation mechanism of INpro.

The droplet sizes investigated in this study (0.5 μl) are larger than atmospheric cloud droplets, which are a few picoliters in volume. Our experimental setup could be modified to analyze smaller droplets with higher surface-to-volume ratios; however, it is limited by the resolution of the optical microscope and the time resolution of the camera. A droplet with 50 μm in diameter would freeze completely within 1 ms of time (*41*, *68*), and the videos would not allow us to identify the onset location.

Another limitation of the instrumentation is the possible interaction of the glass slides with INpro, which was reduced to a minimum with the fluoropel coating. Levitated droplets could be analyzed to exclude all interfaces other than the AWI (*49*), but such instrumentation continues to be limited by the optical lens effect of a spherical droplet, preventing exact views of the onset locations, and by the challenge to control the temperature and relative humidity of the droplets' environment.

### Outlook and implications

The developed methodology can further be applied to investigate the role of the AWI in the heterogeneous ice nucleation of other (bio)molecules and surfactants. Identifying freezing mechanisms at the AWI versus in the bulk of supercooled droplets could help explain how atmospheric droplets freeze. AWI freezing could trap internal water in an ice shell before the droplet shatters due to the pressure from the interior of the droplet. This process could lead to secondary ice production (*72*). Further studies could analyze whether INpro from fungi (*73*, *74*) or other bacteria (*75*) involve the AWI, similarly to Snomax (e.g., inaZ and lipids). Measuring a wide range of chemically different samples could identify the molecular drivers for ice nucleation at the AWI interface. Overall, elucidating the molecular ice nucleation mechanism of (bio)molecules may enable predictive capabilities needed for application in, e.g., cryo-preservation (*4*) and weather modification (*5*), and would lead to a more mechanistic understanding of aerosol-cloud interactions (*7*).

## MATERIALS AND METHODS

### Sample preparation

#### Snomax and filtered Snomax

Snomax was purchased from the manufacturer (Snomax International, USA). Ultrapure water (molecular biological reagent water, Sigma-Aldrich, USA) was added to a dry mass of Snomax to obtain a stock suspension with a concentration of 1 wt %. The pH of the Snomax solution was measured with a pH meter (Orion, Labstar PH111, Thermo Fisher Scientific, USA) to be 6.6, which suggests only a minor influence from the sample matrix on the pH and thus no influence on the ice nucleation ability of INpro (*29*). One sample of 0.1 wt % was filtered with a 220-nm syringe filter (PES membrane, Merck Millipore, USA) to obtain the filtered Snomax sample.

#### Extracted proteins from Snomax

The sample matrix of Snomax was modified by separating polar molecules and lipids from proteins via the Folch extraction procedure, based on (*33*). Methanol [4 ml; high-performance liquid chromatography (HPLC) grade, VWR, USA] were added to 1 ml of a Snomax stock suspension (1 wt %) in a falcon tube. The tube was vortexed before and after the addition of 2 ml of chloroform (HPLC grade, Thermo Fisher Scientific, USA). Phase separation was achieved by adding 3 ml of ultrapure water (molecular biological reagent water, Sigma-Aldrich, USA) followed by centrifugation (3214 rcf, 3 min). The procedure yielded a polar phase on top of a nonpolar phase with an amphiphilic layer of proteins in between the two phases. The middle phase was collected and dried under vacuum to prepare a 1 wt % stock suspension in ultrapure water (molecular biological reagent water, Sigma-Aldrich, USA). Because the material did not dissolve completely and did not create a stable suspension, the sample was centrifuged (3214 rcf, 5 min) to remove large particles. The supernatant was diluted with a factor of 10 and tested for ice nucleation with the high-speed setup. We refer to this sample as lipid-free Snomax.

#### Addition of surfactants (Triton X-100)

Triton X-100 (laboratory grade, Sigma-Aldrich, USA) was added to the Folch extracted Snomax protein fraction to obtain a 0.3 mM Triton X-100 solution that includes INpro. Concentrations above the critical micelle concentration (0.23 mM for Triton X-100) are necessary to dissolve membrane proteins (*66*). Triton X-100 was chosen as a surfactant to conserve ice nucleation activity, which would not be possible when using surfactants that denature proteins (e.g., SDS) (*32*). The solution was sonicated before usage (10 min) to ensure that the proteins were dissolved by the added surfactant. We refer to this sample as surfactant-added Snomax.

#### Cultivated P. syringae

*P. syringae* cells were cultivated similarly to that in (*76*) with the strain 31R1. The culture was grown for 3 days in nutrient broth growth medium (Difco Nutrient Broth, Avantor, USA) at 26°C and shaking at 225 rpm. After the initial culture was grown, the solution containing the cells was diluted with Milli-Q water (resistivity of 18.2 megaohms·cm at 25°C) to reach an optical density of 0.06 at a wavelength of 600 nm, which is equivalent to a cell number concentration of approximately ∼1 × 10^8^ cells ml^−1^, and an ionic strength that is not expected to influence the ice nucleation temperatures.

#### Docosanol

Docosanol solutions were prepared with 1-dososanol (98%, Sigma-Aldrich, USA) in hexane (98.5%, Sigma-Aldrich, USA) instead of water due to the insolubility of 1-docosanol in water. Before the analysis, the solutions in hexane were pipetted on the water droplets, and hexane was evaporated at room temperature for 1 min. The concentration of 1 mM relates to the molar concentration of 1-docosanol on the volume of the water droplet that was investigated.

#### Birch pollen washing water

BPWW was prepared from natural pollen grains sampled from a birch tree of the species *B. pendula* located close to Kitsilano beach in Vancouver, British Columbia, Canada (GPS: 49.272719, −123.157323). The pollen grains were sampled into centrifugal tubes (sterile, 50 ml, Falcon, Fisher Scientific, USA), and the extracts were prepared directly after the sampling process similar to that in (*61*, *64*). A dry mass of the samples was added to a corresponding volume of ultrapure water to obtain a stock suspension/solution with a concentration of 10 wt %. After the extraction was completed (6 hours), the suspension was filtered [220 nm, polyethersulfone (PES) membrane, Merck Millipore, USA] and diluted with a factor of 100 for further measurements.

#### Surface tension measurements

A hanging droplet tensiometer (OCA 15EC, DataPhysics Instruments, Germany) served as the instrument to conduct surface tension measurements. For every experiment, the syringe of the instrument was rinsed six times with Milli-Q water and thereafter filled with the sample solution. Afterward, a droplet with a volume of ∼20 μl was dispensed from a syringe and illuminated with a diffuse light source in a chamber that was tempered to 22°C. A camera mounted on the opposite side took images of the droplet. The corresponding software (SCA202, DataPhysics Instruments, Germany) calculated the surface tension of the droplet by analyzing the shape of the droplet and fitting the Laplace-Young equation (*77*, *78*). Six measurements with different droplets per sample were taken to show the deviation of the analysis due to evaporation effects, geometrical deviations, and temperature fluctuations. Every droplet was left hanging for 60 s to reach an equilibrium state with the surrounding ambient air.

For the 1-docosanol sample, a corresponding amount of a solution in hexane was pipetted onto a hanging droplet with 10 μl. After the hexane was evaporated, more water was dispersed to produce a 20-liter droplet with the 1-docosanol layer on the surface of the droplet. For the surfactant-added Snomax sample, the droplet volume was set to 14 μl to avoid dropping the droplets from the needle due to the low surface tension associated with this sample.

### High-speed cryo-microscopy

#### Setup

For high-speed imaging, a high-speed camera (Chronos 1.4, Krontech, Canada) was mounted onto a microscope (BX, Olympus, Japan) with a cold stage similar to Dymarska *et al*. (*79*). The camera was operated with 2100 frames per second and an 800 × 800 pixel view. The microscope was equipped with a cryo-cell that controlled the temperature (Fig. 1A). The cryo-cell was cooled by a lauda chiller (Proline PR 1290, Lauda-brinkmann, USA) pumping refrigerant (CryoCool, heat transfer fluid, SCC5, Thermo Fisher Scientific, USA) with a temperature of −75°C through a steel block. The set-point temperature was regulated by a heater unit on top of the steel block and measured by a thermocouple, a controller unit (CN16DPT-220, Omega Engineering, USA), and the corresponding software (Platinum software for CNPT series, Omega Engineering, USA). A chamber on top of the heater unit contained a gold-coated silicon wafer as a stage for holding microscopic glass slides. The chamber was sealed air-tight and connected to gas inlets and outlets. A droplet with 0.5 μl was squeezed between two fluoropel-coated glass cover slides (see Fig. 1B for a detailed view). Another piece of glass cover served as a spacer between the two glass slides, separating them by 220 μm. Microscopic glass cover slides (12 mm, BoliOptics, USA) were covered with fluoropel (Fluoropel 800, 0.2%, Cytonix, USA), a fluoroacrylic polymer that provides a hydrophobic and oleophobic interface, repelling (bio)organic molecules such as INpro (*50*, *51*). We refer to the fluoropel-coated glass slide as a protein-repellent slide. The glass slides were cleaned with acetone and Milli-Q water and thereafter dried at 100°C in an oven for 15 min. Afterward, the slides were dipped into the fluoropel solution with tweezers and held for 10 s. Last, the glass slides were carefully removed from the solutions and dried in an oven at 100°C for at least 2 hours. Other glass slides were used to probe the role of the slide-INpro interactions (fig. S8), including a siliconized slide (12 mm, siliconized glass circle cover slides, Hampton Research, USA).

#### Freezing experiment

To perform an experiment, 0.5 μl of sample solution was pipetted onto a fluoropel-coated glass slide and squeezed between another glass slide separated by spacers (smaller glass slides with 220-nm thickness). This assembly yielded cylindrical shaped droplets with a diameter of around 1.5 mm. The AWI became clearly visible with the microscopic unit as a dark circle (see Fig. 2 and figs. S10 and S7 for examples). The closed cryo-cell was flushed with dry N_2_ gas for 10 s to reduce the relative humidity of the surrounding air and avoid condensation issues within the optical beam path. Thereafter, the droplet was rapidly cooled to 0°C and incubated at that temperature for 1 min to equilibrate the gas phase with water vapor in that temperature regime. Afterward, the droplet was cooled by a rate of −3°C min^−1^ until freezing was observed optically. We did not observe an influence of water vapor condensation on changing the size of the droplet while it was cooled, suggesting that the water vapor equilibrium at 0°C was sufficient. The low deviation of the freezing temperatures between the independent runs (figs. S1 and S3) and the random scattering of nucleation locations within the bulk as observed for BPWW and *P. syringae* cells (Fig. 3, B and G) indicate that the temperature is uniform within the assembly. The nucleation temperature for Snomax changes only slightly when modifying the freezing rate (*45*), and, therefore, the freezing behavior of inaZ when cooled with −3°C min^−1^ represents an atmospherically relevant freezing scenario. Simultaneously to the cooling cycles, the high-speed video recordings were started. Whenever the droplet froze, the brightness decreased notably, and the video recording was stopped manually. The freezing temperature was then noted. Last, the video section that contained the freezing process was stored and evaluated.

#### Statistical considerations

Because of the stochastic nature of ice nucleation (*1*), repeated freezing experiments with the same sample solutions can have various outcomes (e.g., different onset locations and temperatures). Only experiments with multiple droplets can capture the ice nucleation spectra completely (*80*). Therefore, a single experiment is not sufficient to investigate a freezing mechanism because freezing could have occurred at or close to the AWI just by chance. We conducted Monte Carlo simulations (fig. S6) to estimate the number of experiments necessary to differentiate between ice nucleation in the bulk and ice nucleation influenced by the AWI.

#### Temperature validation

To test the temperature accuracy and precision of the cryo-microscope stage, we measured two samples (Snomax of 10^−3^ wt % and BPWW) with the cryo-microscope and with the FINC, which is described in detail elsewhere (*42*). In short, FINC is a high-throughput droplet freezing assay, which measures 288 droplets per run in polymerase chain reaction trays (96-well Piko PCR Plates, SPL0960, Thermo Fisher Scientific, USA) cooled with an ethanol bath. The freezing is detected with an optical camera because the freezing of droplets changes their optical density. The temperature accuracy and uncertainty of FINC were thoroughly determined during the development phase of the instrument (*42*). Droplet volumes of 10 μl were used for the FINC measurements. To account for the difference in droplet size between the two instruments, the samples were diluted with a factor of 20 ensuring the same mass of ice nucleating substance per droplet. The temperature ramp of FINC was −1°C min^−1^. Figure S2 shows a high agreement between the freezing spectra measured with FINC (288 droplets) and the same sample measured with 32 droplets on the cryo-microscopic cold stage confirming that the temperature measured with the cryo-microscope represents the freezing temperatures of the samples.

#### Data evaluation

For data evaluation, we used ImageJ (National Institutes of Health, USA) and calculated the polar coordinates of the ice embryos' origin. First, a snapshot was taken from the high-speed videos when the ice crystal was still small enough to locate its onset location (Fig. 2 and figs. S7 and S10). Thereafter, a macro script within ImageJ helped to calculate the polar coordinates of the ice nucleation onset location. Last, the processed image and the coordinates (angle and distance) of the nucleation event in regard to the center of the droplet and with the droplets’ radius normalized to 1 were stored (see Fig. 2 and figs. S7 and S10 for examples).

## Supplementary Materials

### This PDF file includes:

Suplementary Text Figs. S1 to S12 Table S1 Legends for movies S1 to S9
