# Supplementary material for: High-speed cryo-microscopy reveals that ice-nucleating proteins of Pseudomonas syringae trigger freezing at hydrophobic interfaces
Source: Sci Adv. 2024 Jul 3;10(27):eadn6606. doi: 10.1126/sciadv.adn6606 (PMC11221516; doi:10.1126/sciadv.adn6606)
Supplement: Supplementary file 1 — Suplementary Text Figs. S1 to S12 Table S1 Legends for movies S1 to S9 [file sciadv.adn6606_sm.pdf]

Science Advances
AAAS

Supplementary Materials for

High-speed cryo-microscopy reveals that ice-nucleating proteins of *Pseudomonas syringae* trigger freezing at hydrophobic interfaces

Paul Bieber and Nadine Borduas-Dedekind

Corresponding author: Paul Bieber, pbieber@chem.ubc.ca; Nadine Borduas-Dedekind, borduas@chem.ubc.ca

Sci. Adv. **10**, eadn6606 (2024)
DOI: 10.1126/sciadv.adn6606

The PDF file includes:

Suplementary Text
Figs. S1 to S12
Table S1
Legends for movies S1 to S9

Other Supplementary Material for this manuscript includes the following:

Movies S1 to S9

Freezing temperatures

We analyzed the freezing temperatures of Snomax (Snomax international, USA) in solutions of different mass concentrations of 10^{-1} wt%, 10^{-3} wt%, and 10^{-5} wt%, docosanol (98%, Sigma-Aldrich, USA), birch pollen washing water (BPWW) (similar to ref (*64*)), filtered Snomax, lipid-free Snomax, surfactant-added Snomax, filtered Snomax on siliconized glass slides, filtered Snomax in a halocarbon oil matrix, intact *Pseudomonas syringae* cells (similar to ref (*76*)), and the handling blank, which consists of ultrapure water (molecular biological reagent water, Sigma-Aldrich, USA) that was treated with identical labware as the other samples (see Methodology for additional information). The handling blank of the cultivation procedure was measured by Worthy et al.,(*76*) and is clearly below the freezing temperatures observed for *P. syringae* cells. Samples were added to the cryo-chamber as an aqueous droplet (0.5 µL), and every droplet was frozen and thawed four times and analyzed with the high-speed cryo-microscope. After four cycles, most droplets evaporated too much water to continue freeze-thaw cycles with neglecting the effect of smaller volumes (e.g. the mean reduction of droplet sizes due to evaporation for all 8 droplets of the Snomax (10^{-3} wt%) sample was 15% during 4 freeze-thaw cycles). Therefore, a fresh droplet was used after 4 freeze-thaw cycles for a total number of 32 observations per sample. The fraction of frozen droplets, $f_f(T)$, was calculated using

$$f_f(T) = \frac{n(T)}{n_{total}} \quad \text{(S1)}$$

where $n(T)$ is the cumulative count of freezing events at a certain temperature T and n_{total} it the total number of observed frozen droplets at –30°C (n_{total} = 32). Figure S1 shows the freezing spectra of all analyzed samples.

Temperature comparison with the drop freezing ice nuclei counter (FINC) instrument

To ensure that our freezing temperatures measured by the cryo-microscopy setup were statistically significant, we made measurements with our drop freezing ice nuclei counter (FINC) (*42*). The freezing temperatures measured with the cryo-microscope (32 data points) are similar to the temperatures measured with FINC (288 data points) (see overlapping $f_f(T)$ values in Figure S2). For Snomax (10^{-3} wt%) the T_{50} of the cryo-microscope measurement was –6.5 °C, compared with –6.6 °C obtained from the FINC measurements. For BPWW, we measured the T_{50} to be –14.7 °C, compared with a T_{50} of –14.9 °C. The difference between the T_{50} values is smaller to the uncertainty of the FINC instrument ($\pm$ 0.5 °C) (*42*). Therefore, we conclude that the cryo-microscopy experiments provide accurate measurements of the freezing temperatures for the samples. Furthermore, we can exclude the possibility of insufficient sampling of the temperature as analyzed by ref (*80*) in our system.

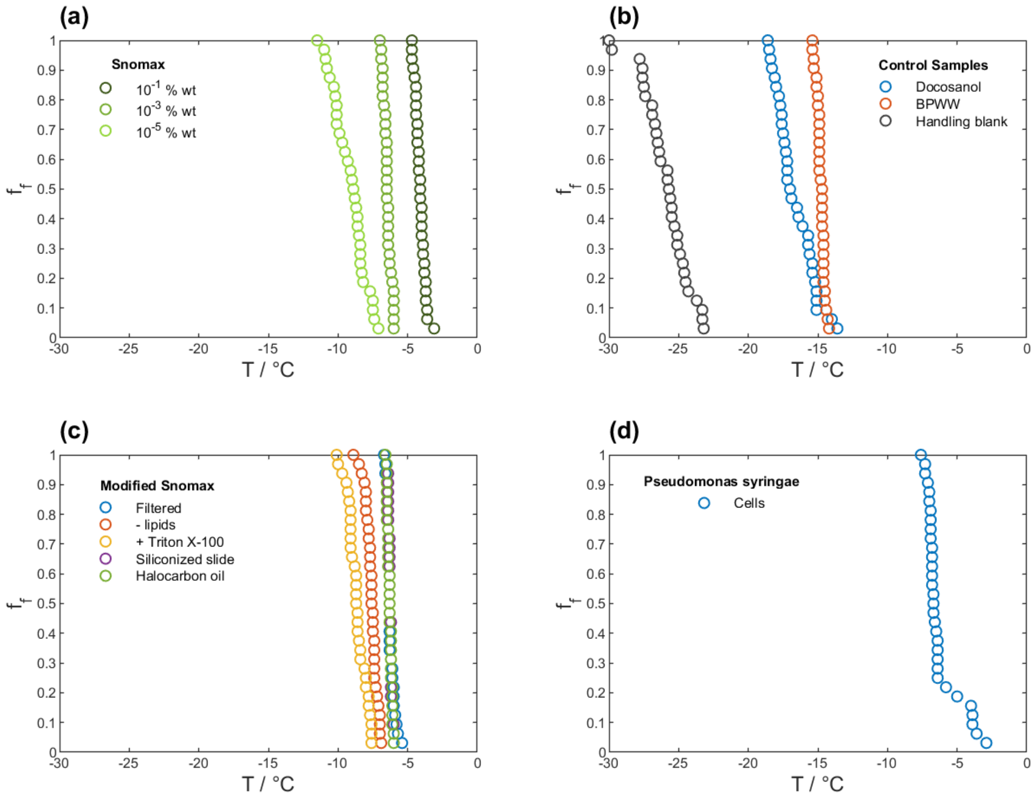

Fig. S1: Frozen fractions (f_f) against the temperature (T) for all analyzed samples. (a) Snomax in different mass concentrations, (b) docosanol-covered droplet, birch pollen washing water (BPWW), and the handling blank as control samples, (c) filtered Snomax, lipid-free Snomax, surfactant-added Snomax, filtered Snomax on a siliconized slide, and filtered Snomax in a halocarbon oil matrix, and (d) intact *P. syringae* cells.

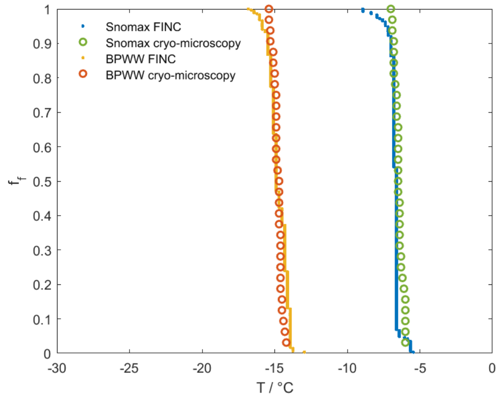

Fig. S2: Frozen fractions (f_f) plotted against the temperature (T) for a temperature comparison of the Snomax (10^{-3} wt%) and BPWW samples between the cryo-microscope (32 droplets) and the FINC instrument (288 droplets). Note that for the FINC measurements the samples were diluted 20 fold to account for the 20 times larger droplet volumes. Therefore, the mass of ice nucleation active material in the droplets is equal.

Effects of the freeze-thaw cycles on the freezing temperature and locations

Next, we estimated the effect of the freeze-thaw cycles on the freezing temperatures. We plotted the recorded temperatures for all 8 droplets against the number of the freeze-thaw cycles (Figure S3). We did not find any trend in ice nucleation activity with repeated freeze-thaw cycles for Snomax aqueous solutions (Figure S3a, b, c, f, g, h, i, and j). This behaviour indicates that the 4 freeze-thaw cycles did not modify the ice nucleation ability of Snomax. The docosanol sample showed some variations in temperatures, however no correlation between the freeze-thaw cycles and the freezing temperatures was observed (Figure S3d). We attribute the variability to the difficulty in reproducing the docosanol layer on the water droplet. Furthermore, the freezing temperatures were stable for BPWW (Figure S3e) and varied only slightly for the cultivated *P. syringae* sample (Figure S3k). Overall, all samples were stable during the freeze-thaw experiments and thus, the 32 observations were pooled and one freezing temperature is reported for each experiment (Table S1). The 32 freezing temperatures were then used to plot Figure 3 and S8.

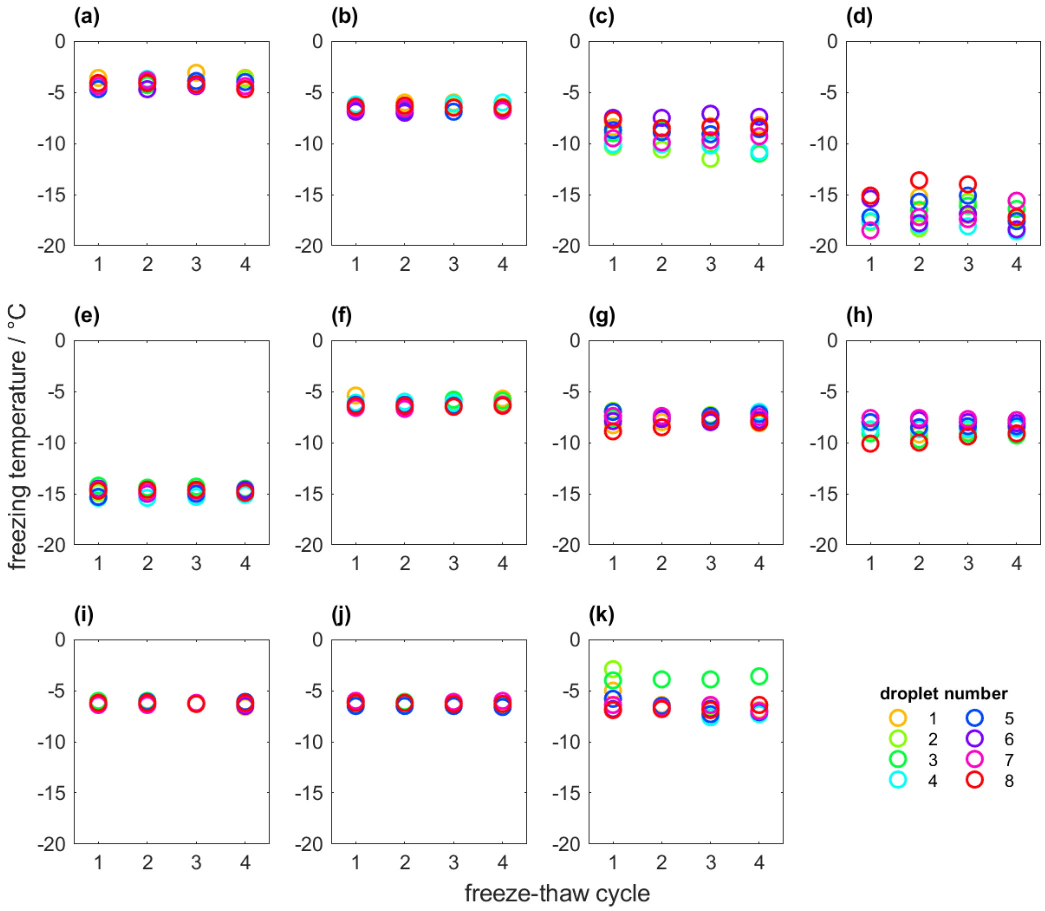

Fig. S3: Recorded freezing temperatures over four freeze-thaw experiments. (a) Snomax 10^{-1} wt%, (b) Snomax 10^{-3} wt%, (c) Snomax 10^{-5} wt%, comparison samples (d) docosanol-covered aqueous droplet and (e) birch pollen washing water, (f) filtered Snomax, (g) lipid-free Snomax, (h) surfactant-added Snomax, (i) filtered Snomax on a siliconized slide, (j) filtered Snomax in a halocarbon oil matrix, and (k) intact *P. syringae* cells.

Furthermore, the repeated freeze-thaw cycle did not have a noticeable influence on the onset locations. In fact, we calculated the normalized volume fraction (v_f) of the nucleation event by assuming cylindrical geometry of the droplet with constant height (h) and thus using

$$v_f = \frac{v_{nucleation}}{v_{total}} = \frac{r^2_{nucleation}\pi h}{r^2_{total}\pi h} = \left(\frac{r_{nucleation}}{r_{total}}\right)^2 \tag{S2}$$

where $v_{nucleation}$ is the volume within the nucleation event occurred, v_{total} is the total volume of the droplet, $r_{nucleation}$ is the distance from the center of the droplet to the location of the nucleation event, and r_{total} is the distance from the center of the droplet to the AWI (i.e. the droplet's radius). We identified that BPWW, *P. syringae* cells, lipid-free Snomax, and Snomax in the halocarbon oil matrix (Figure S4e, j, g, and k) scatter randomly across the volume, whereas all other samples (Figure S4a, b, c, d, f, h, and i) showed at least some affinity for the AWI (i.e. v_f close to 1). We did not observe an obvious trend of the behaviour with freeze-thaw cycles confirming the stability of the analyzed samples during the experiments.

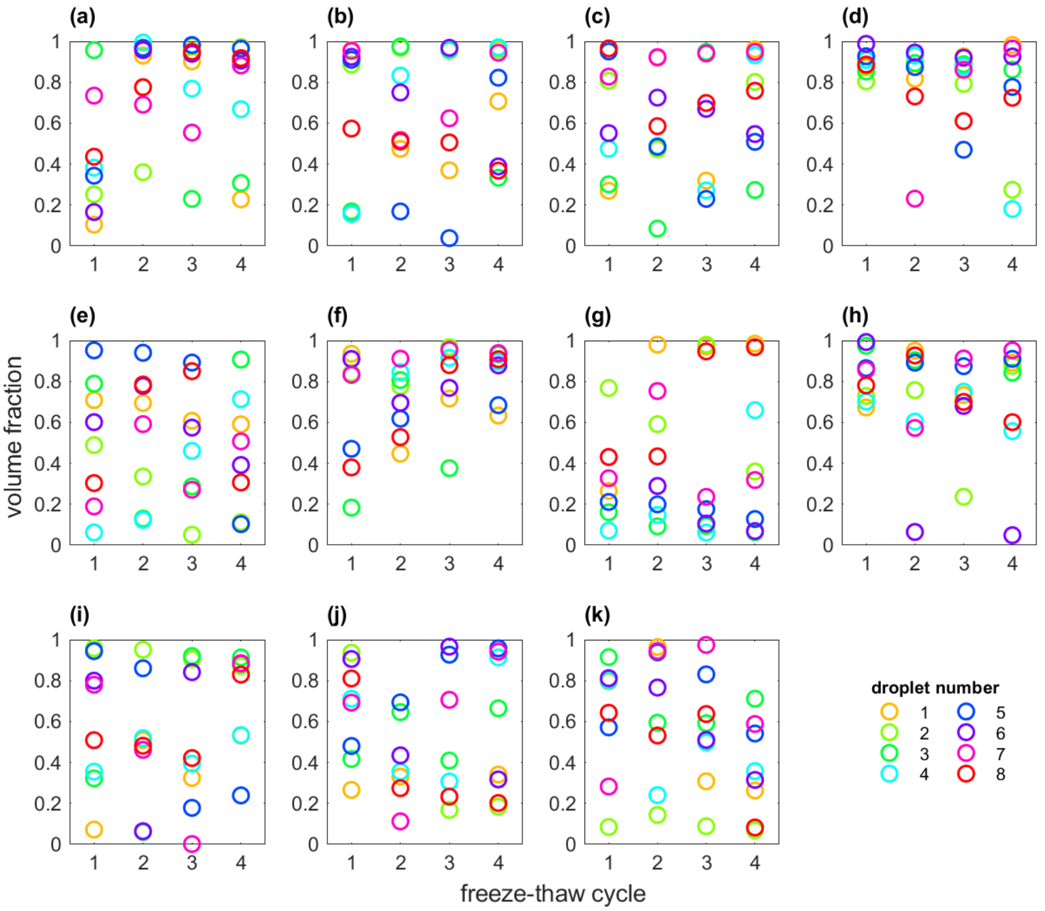

Fig. S4: Volume fraction over four freeze-thaw experiments. (a) Snomax 10^{-1} wt%, (b) Snomax 10^{-3} wt%, (c) Snomax 10^{-5} wt%, comparison samples (d) docosanol-covered aqueous droplet and (e) birch pollen washing water, (f) filtered Snomax, (g) lipid-free Snomax, (h) surfactant-added Snomax, (i) filtered Snomax on a siliconized slide, (j) filtered Snomax in a halocarbon oil matrix, and (k) intact *P. syringae* cells.

Geometrical considerations

To quantify the freezing location onset based on the high-speed videos, the volumes of the pancake-shaped droplets were divided into five equal volume parts. First, the radii were normalized to 1, although the width of the droplets were similar as they all had a volume of 0.5 μL. Then, we separated the volumes into cylinders with the radii $r_1 = \sqrt{1/5}$, $r_2 = \sqrt{2/5}$, $r_3 = \sqrt{3/5}$, $r_4 = \sqrt{4/5}$, and $r_5 = \sqrt{5/5}$ (see Figure S5). Thus, the volumes of the intersections are equal, since $V_1 = r_1^2 \pi h = (1/5)\pi h$ and $V_2 = r_2^2 \pi h - r_1^2 \pi h = (2/5 - 1/5)\pi h = (1/5)\pi h$ and so on. We assume that the small vertical curvature present at the AWI as the droplet is squished between the two glass slides is accounted for in the fifth of the volume. This geometrical separation of the volumes allows us to find nucleation events in every volume with a 20% chance if the nature of freezing originates from a volume-dependent process. Furthermore, nucleation events at the AWI increase the frequency of nucleation hits in the outermost sector. The distance between r_5 and r_4 is larger than the resolution of detecting an early stage ice crystal. Therefore, we can identify AWI-dependent freezing behaviour for samples with enhanced freezing activity at the outermost sector.

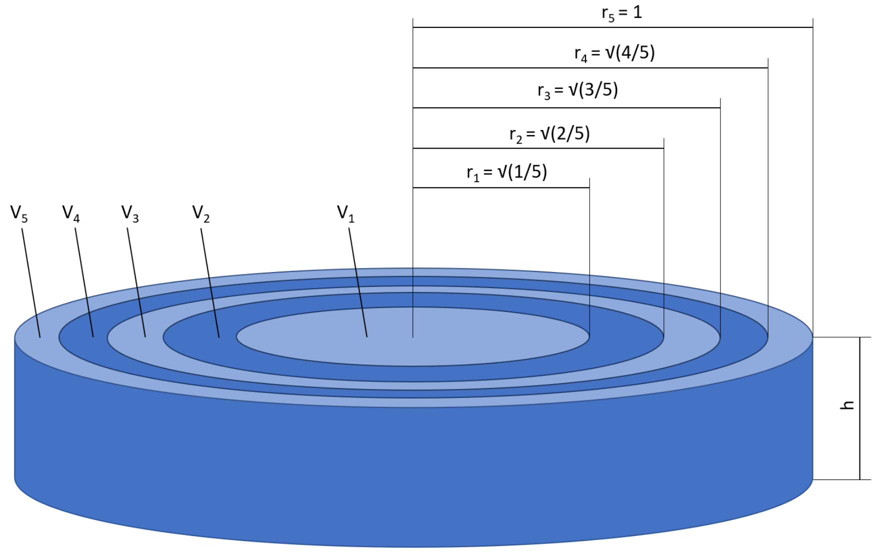

Fig. S5: Schematic drawing of the geometrical separation of a droplet into five equivalent volumes. The height of each cylindrical droplet is 220 μm (see Figure 1).

Monte Carlo simulations

To estimate how many experiments are necessary to differentiate between freezing at the AWI or in the bulk, we conducted Monte Carlo simulations. We simulated the process of bulk nucleation by randomly distributing onset locations in the volume of the droplet (separation of the volume shown in Figure S5). First, a random number between 0 and 1 was generated and compared with 0.2, representing 20% chance to fall within a fifth of the droplets volume (i.e. outermost sector V_5). Second, we repeated this process n times to calculate the frequency of nucleation in the interfacial sector, by counting the positive hits of a run and dividing by n. This simulation was then repeated 1000 times to calculate a distribution (Figure S6a, right hand graph for each n value). The first 30 runs, representative for different outcomes in the 1000 simulations, and the probability density of all 1000 simulations with n experiments are

shown in Figure S6a. We considered that $n = 32$ was a good balance between statistically eliminating the possibility of coincidence of nucleation at the AWI and experimental manipulation and experimental time.

Furthermore, we simulated possible outcomes of different experiments. Every experiment contains 32 observations and nucleation onset locations are simulated with different assumptions. The results are plotted in target plots (Figure S6b), that show the polar coordinates of nucleation onset locations and the nucleation frequency in each sector from 0 (no nucleation events) to 1 (all nucleation events in this sector) as a color code from yellow to red. First, we simulated volume dependent nucleation with generating random polar-coordinates (Figure S6b). Furthermore, we simulated results for a probability of 50% and 100% for nucleation at the AWI (Figure S6b). Clearly, we can differentiate between bulk freezing and freezing that is influenced by nucleation at the AWI by more than 50% with $n = 32$ observations per sample.

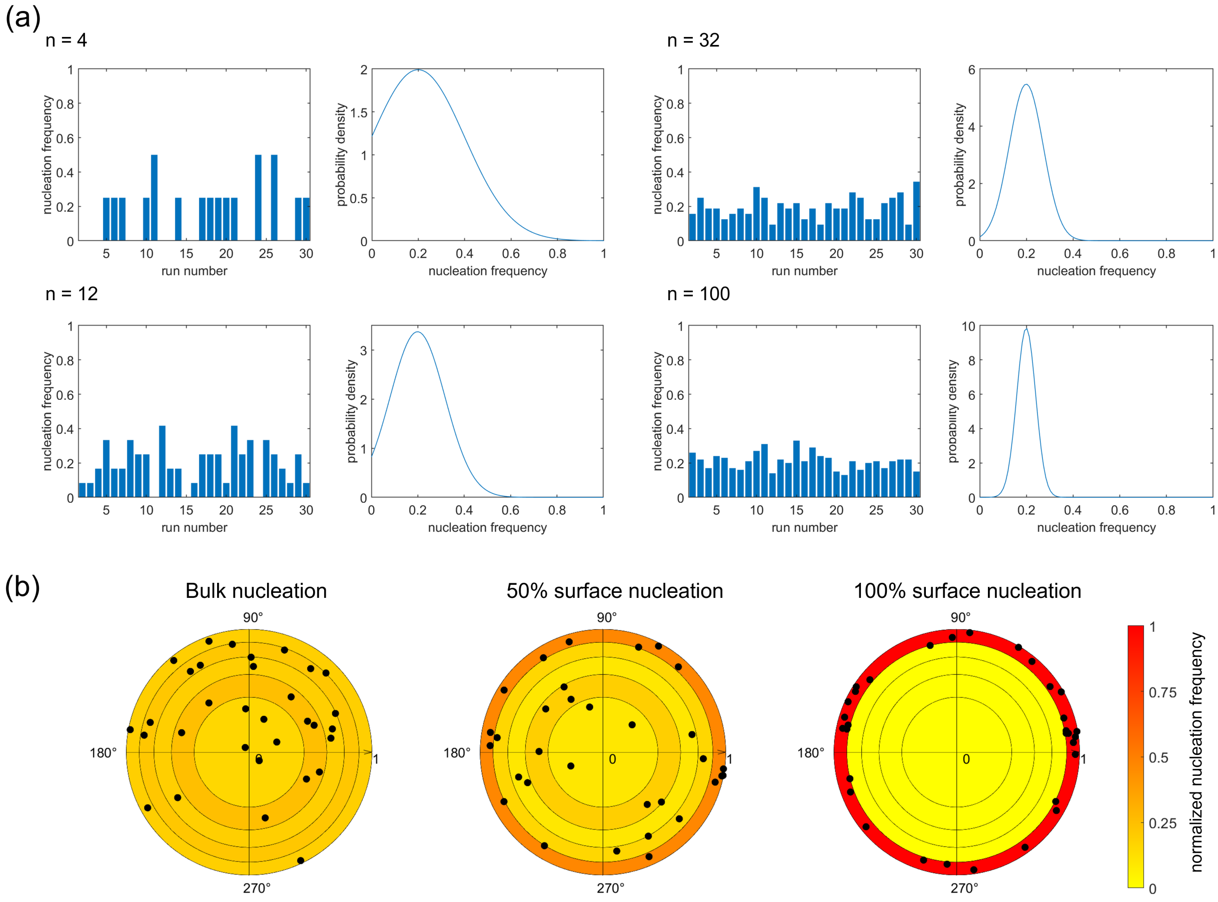

Fig. S6: Monte Carlo simulations of the statistical scattering of freezing immersed in the droplet. (a) The simulations were run for a probability of 20% that freezing occurs in the outermost sector of the droplet (which corresponds to a fraction of 0.2 of the total volume). 1000 simulation runs were performed by varying the number of experiments per sample, referred to as n. The left diagrams show the nucleation frequency at the interfacial sector (AWI) of the first 30 runs. The right diagrams show the Gaussian distribution of the nucleation frequency at the interfacial sector. (b) Monte Carlo simulations for different experimental outcomes. Simulation of volume freezing by generating a random number i between 0 and 1 and calculating the radii with $r = \sqrt{i}$, simulating a second random number j and calculating the angel with $\psi = 2\pi j$, and drawing the polar coordinates. Additional target plots with the probability of 50% and 100% to detect nucleation at the AWI are to visualize the statistical significance of freezing at the AWI.

Additional high-speed images of INpro samples

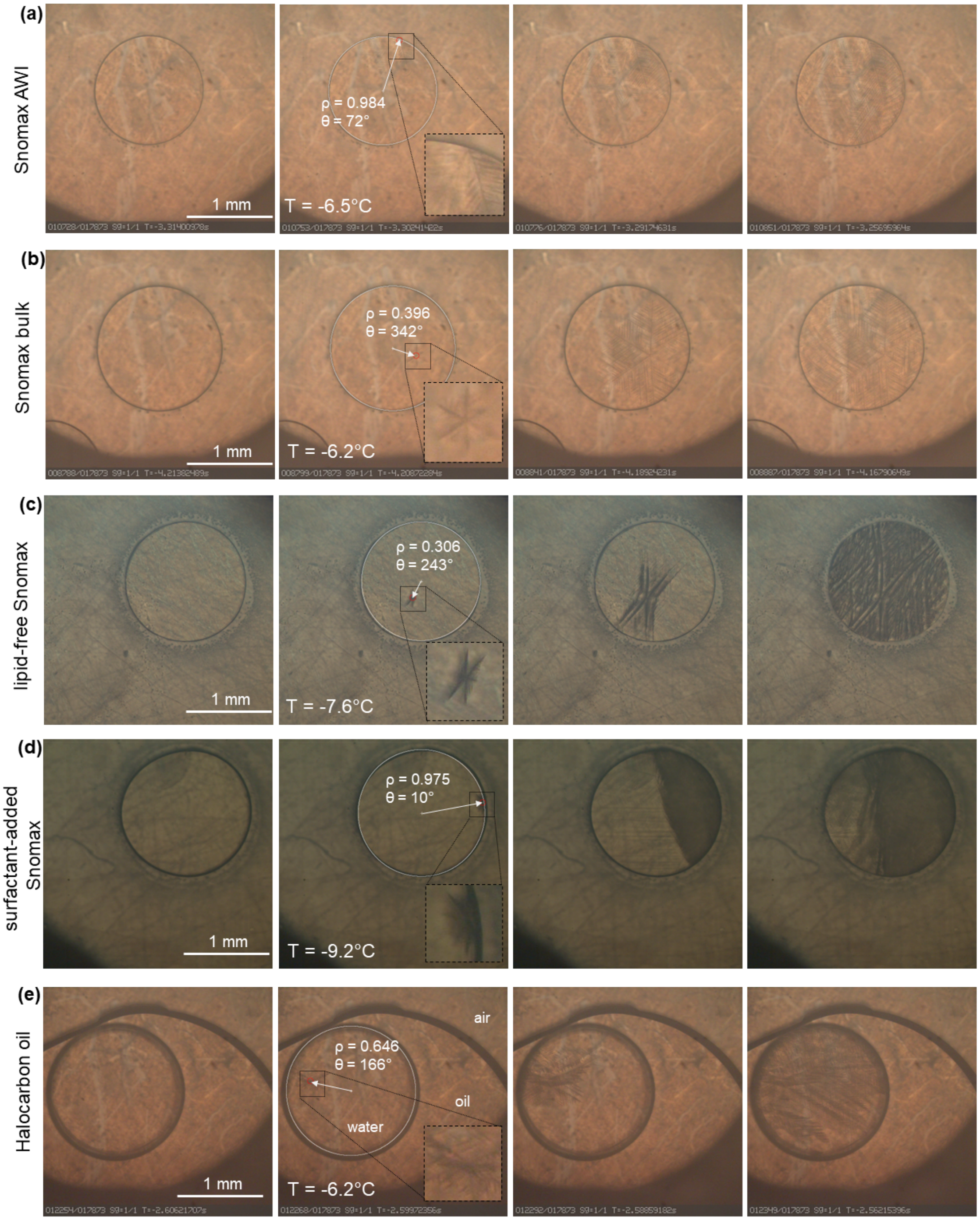

Fig. S7: Additional example images of freezing. Snomax with the concentration 10^{-3} wt% nucleating (a) at the AWI and (b) in the bulk. (c) Folch extracted Snomax sample (lipid-free) nucleating in the bulk and (d) the same sample with added surfactants (Triton X-100, 0.3 mM) nucleating at the AWI. (e) Filtered Snomax in the halocarbon oil emulsion. Note that the color settings of the camera were different for the images in (c) and (d).

Ice nucleation locations

In addition to the results shown in the manuscript (Figure 3), we measured the ice nucleation onset locations for a Snomax dilution series (10^{-1} wt%, 10^{-3} wt%, and 10^{-5} wt%) as well as filtered Snomax on siliconized glass slides and in a halocarbon oil emulsion (Halocarbon 6.3 Oil, Halocarbon^{TM}, USA). The corresponding target plots are shown in Figure S8. The enhancement of nucleation by INpro at the AWI is more pronounced with the more concentrated Snomax samples (Figure S8a and b) compared to the least concentrated one (Figure S8c). More INpro adsorb to the glass interface, when the fluorpel slide for a filtered snomax sample (Figure 3) is replaced by a siliconized slide (Figure S8d). Furthermore, the replacement of the air and glass interfaces by halocarbon oil leads to a random distribution of nucleation onset locations across the droplet, because the entire droplet is surround by the same interface (Figure S8e).

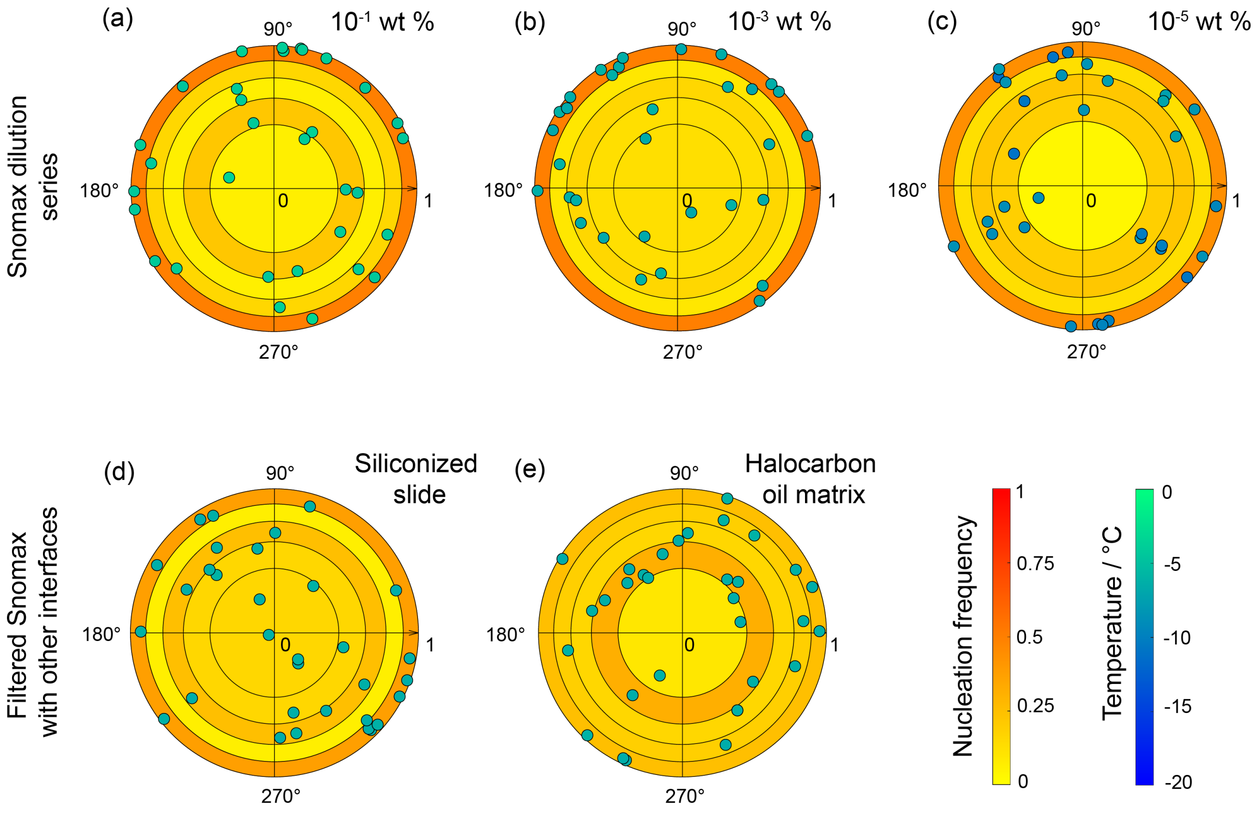

Fig. S8: Additional target plots for aqueous droplets of Snomax in different concentrations. (a) 10^{-1} wt%, (b) 10^{-3} wt%, and (c) 10^{-5} wt%. Target plots for the filtered Snomax sample measured on (d) siliconized glass slides and (e) in an oil emulsion.

Temperature dependency of the nucleation location

We plotted the location of the nucleation events (normalized to a radius of 1) as function of the freezing temperature to identify possible correlations (Figure S9). Clearly, in the investigated samples containing INpro, the freezing temperature is independent of the freezing location. This control further supports that the properties of INpro and the sample matrix are responsible for the ice nucleation at the AWI in every experiment.

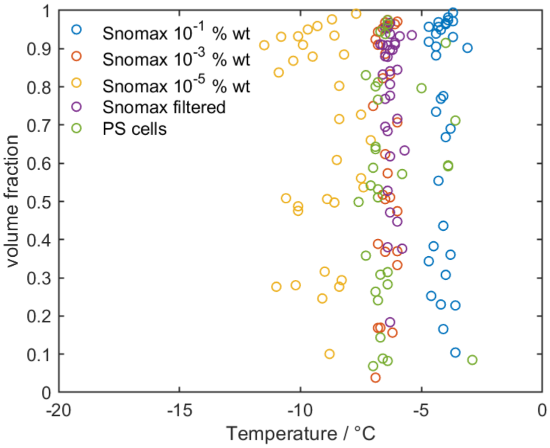

Fig. S9: Volume fraction of the nucleation event against the temperature for different samples of Snomax and *Pseudomonas syringae* (PS) cells.

High-speed images of the control samples

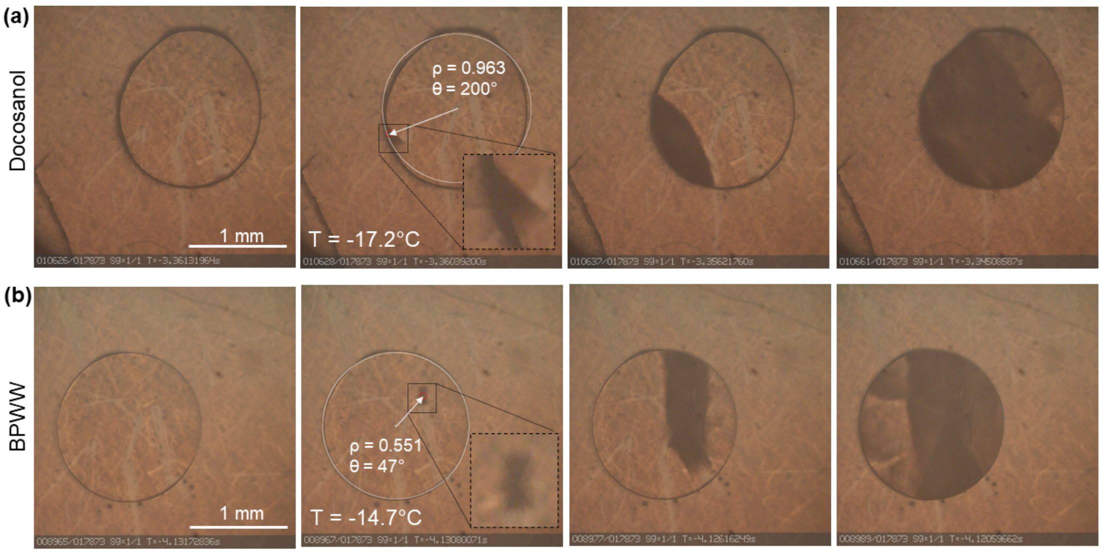

Fig. S10: Example images of the control samples. (a) A droplet coated with docosanol freezing at the AWI and (b) BPWW freezing in the bulk.

Ice propagation velocity

The propagation velocity of the ice phase was evaluated from high-speed videos. We took screenshots from three to five videos of different samples at two time points (one shortly after the nucleation of ice and one after the crystals propagated in about a third of the investigated volume). Thereafter we subtracted screenshot 1 from screenshot 2 and converted the image to a negative image (example in Figure S11). The distance of propagation d was measured using imageJ (National Institutes of Health, USA) and the distance was converted to mm using the relationship of 1.00 mm = 297 pixel obtained from an optical caliper. From the time markers of the two images we are able to calculate the time interval, t, and thereafter the velocity of ice crystal propagation, v, using

$$v = \frac{d}{t} \tag{S3}$$

and by assuming linear propagation velocities. The resulting values are given in Figure S12. Clearly, lower ice nucleation temperatures led to faster propagation of the ice phase. This trend is consistent with literature values for free growing dendrites and typical for the kinetically unhindered crystal growth regime (*41*, *68*).

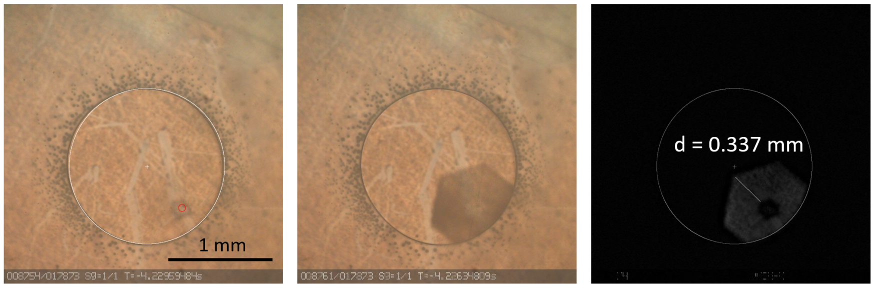

Fig. S11: Example of evaluating ice propagation velocities from a sample containing birch pollen washing water extracts. The time stamp of the first image is 4.229595 s, whereas the second image was taken at 4.226348 s before the high-speed recording was stopped, which results in a time interval of 3.25 ms. The distance of the propagating ice front was measured to be 0.337 mm resulting in a propagation velocity of 10.4 cm s^{-1}.

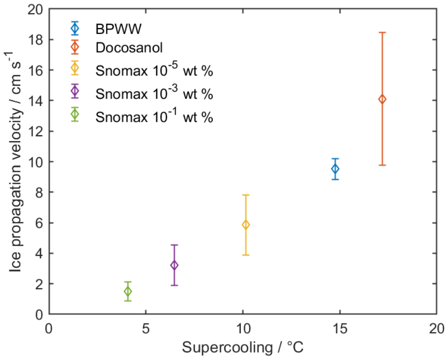

Fig. S12: Calculated ice propagation velocities for various samples. Five individual videos were evaluated for samples of birch pollen washing water extracts (BPWW), and docosanol, whereas three videos for each of the Snomax samples. The mean values of the individual measurements are shown as diamonds and standard deviations as whiskers. The temperature interval of one sample type was kept at 0.3 °C.

Tab. S1: Summary table of the investigated samples. Table includes the names and concentrations of all analyzed samples, the median nucleation temperature (T_{50}) $\pm$ the standard deviation (SD) of 32 freezing events, the nucleation frequency in the superfacial sector of the droplet, and the surface tension $\pm$ SD of six pendant droplet measurements.

Sample (Concentration)	T_{50} $\pm$ SD / °C	Interfacial nucleation frequency / %	Surface tension $\pm$ SD / mN m^{-1}
Handling blank	-25.8 $\pm$ 1.7	n/a	72.2 $\pm$ 0.2
Snomax (10^{-1} wt%)	-4.1 $\pm$ 0.4	50	56.2 $\pm$ 0.9
Snomax (10^{-3} wt%)	-6.5 $\pm$ 0.3	50	72.1 $\pm$ 0.2
Snomax (10^{-5} wt%)	-9.0 $\pm$ 1.2	44	72.0 $\pm$ 0.1
Snomax filtrate (10^{-1} wt%)	-6.3 $\pm$ 0.3	59	64.1 $\pm$ 0.9
Lipid-free Snomax (10^{-1} wt%)	-7.6 $\pm$ 0.4	19	72.0 $\pm$ 0.5
Surfactant-added Snomax (10^{-1} wt%)	-8.7 $\pm$ 0.7	47	33.2 $\pm$ 0.2
Snomax filtrate (10^{-1} wt%) on siliconized glass	-6.3 $\pm$ 0.1	38	n/a
Snomax filtrate (10^{-1} wt%) in halocarbonoil matrix	-6.3 $\pm$ 0.2	25	n/a
BPWW (10^{-1} wt%)	-14.8 $\pm$ 0.3	16	72.2 $\pm$ 0.5
Docosanol (1 mM)	-17.1 $\pm$ 1.3	72	58.7 $\pm$ 3.2
Pseudomonas syringae cells ($\sim 1*10^{8}$ cells mL^{-1})	-6.7 $\pm$ 1.2	25	70.6 $\pm$ 0.1

Supplementary high-speed videos

Mov. S1: Snomax sample nucleating ice at the air-water interface (Figure 2a).

Mov. S2: *P. syringae* cells nucleating ice in the bulk of a droplet (Figure 2b).

Mov. S3: Snomax sample nucleating ice at the air-water interface (Figure S7a).

Mov. S4: Snomax sample nucleating ice in the bulk (Figure S7b).

Mov. S5: Lipid-free Snomax sample (Figure S7c).

Mov. S6: Surfactant-added Snomax (Figure S7d).

Mov. S7: Snomax in oil matrix (Figure S7e).

Mov. S8: Docosanol nucleating ice at the air-water interface (Figure S10a).

Mov. S9: Birch pollen washing water nucleating ice in the bulk (Figure S10b).
